# Tip-Enhanced Raman Scattering Imaging of Two-Dimensional Tungsten Disulfide with Optimized Tip Fabrication Process

**DOI:** 10.1038/srep40810

**Published:** 2017-01-13

**Authors:** Chanwoo Lee, Sung Tae Kim, Byeong Geun Jeong, Seok Joon Yun, Young Jae Song, Young Hee Lee, Doo Jae Park, Mun Seok Jeong

**Affiliations:** 1 Department of Energy Science, Sungkyunkwan University (SKKU), Suwon 16419, Republic of Korea; 2 Center for Integrated Nanostructure Physics, Institute for Basic Science (IBS), Suwon 16419, Republic of Korea; 3 SKKU Advanced Institute of Nanotechnology (SAINT), Sungkyunkwan University, Suwon 16419, Republic of Korea; 4 Department of Physics, Sungkyunkwan University, Suwon 16419, Republic of Korea; 5 Department of Physics, Hallym University, Hallymdaehakgil 1, Chuncheon 24252, Republic of Korea

## Abstract

We successfully achieve the tip-enhanced nano Raman scattering images of a tungsten disulfide monolayer with optimizing a fabrication method of gold nanotip by controlling the concentration of etchant in an electrochemical etching process. By applying a square-wave voltage supplied from an arbitrary waveform generator to a gold wire, which is immersed in a hydrochloric acid solution diluted with ethanol at various ratios, we find that both the conical angle and radius of curvature of the tip apex can be varied by changing the ratio of hydrochloric acid and ethanol. We also suggest a model to explain the origin of these variations in the tip shape. From the systematic study, we find an optimal condition for achieving the yield of ~60% with the radius of ~34 nm and the cone angle of ~35°. Using representative tips fabricated under the optimal etching condition, we demonstrate the tip-enhanced Raman scattering experiment of tungsten disulfide monolayer grown by a chemical vapor deposition method with a spatial resolution of ~40 nm and a Raman enhancement factor of ~4,760.

Fabrication of an ultrasharp metal nanotip is essential in various applications and state-of the-art instruments that are used in methods such as tip-enhanced Raman spectroscopy^1^,^2^, scattering-type near-field scanning optical microscopy (s-NSOM)^3^, scanning tunneling microscopy^4^,^5^, and ultrafast electron emission^6^,^7^,^8^. These ultrasharp metal nanotips enable high-resolution topographic imaging up to a few nanometers^1^,^3^,^9^,^10^,^11^ by virtue of the ultrasmall diameter (a few nanometers) of the tip apex. Additionally, the optical signal enhancement and spatial resolution, which are associated with the huge field enhancement and high field confinement, are also improved when such tips are used; this is particularly crucial when these nanotips are applied in instruments such as a tip-enhanced Raman spectroscope and in s-NSOM for chemical and energetic analysis of nanostructures such as nanowires^12^, carbon nanotubes^1^,^11^,^13^,^14^, graphene^15^,^16^,^17^,^18^, and even single molecules^9^,^11^. Also, a control of cone angle of the nanotip is desirable to meet maximum Raman enhancement^11^.

Electrochemical etching is not only widely applied in the fabrication of ultrasharp metal nanotips but is also regarded as the most efficient method for fabricating a tip with an apex radius of a few tens of nanometers. Furthermore, electrochemical etching has advantages over other methods such as the pulling method^19^,^20^,^21^,^22^,^23^ in terms of a high yield, low cost, and short fabrication time. Other method reported by Johnson *et. al.*^11^ which have shown a fabrication of pyramidal tip with a capability of mass production and with a high reproducibility compared to the electrochemical etching. However, such tips are hardly applicable in top-illuminating optical configuration, which is necessary to an opaque sample. The electrochemical etching method involves the use of an electrolyte such as hydrochloric acid (HCl)^13^,^24^. A direct current or an alternating current is applied to a metal wire, and electrically induced oxidation of metal via electron exchange with ions in solution etches the metal surface, ultimately forming a sharp edge. These processes are dominant in the meniscus of the solution around the metal wire owing to the high ion concentration, which is a key phenomenon for shaping the metal nanotip through electrochemical etching^25^.

However, when the chemical process is used, precise control of the ambient conditions (the temperature, humidity, etc.), applied bias, and ion concentration of the etching solution is required to fabricate a tip with the desired shape. Hence, a systematic study on the effect of the applied bias and ion concentration, which are related to the shape of the tip, should be performed. A series of studies has reported on the influence of the viscosity^26^ and the effect of the bias voltage^27^, including its amplitude^25^ and duty cycle^20^. Although a study about controlling the concentration of HCl solution using two different alcohols has been published^24^, it is essential to use only one control factor; this practice plays a key role in precisely controlling the concentration of ions in the etchant. In addition, a study on how the concentration of ions in the etchant determines the radius of curvature (*R*_c_) of etched tips has not been conducted to date. Moreover, a number of previous reports of the electrochemical etching method to fabricate the gold nanotip for tip-enhanced Raman scattering (TERS) presented the reproducibility and estimated the applicability but did not present TERS images^19^,^20^,^21^,^23^,^24^,^26^,^27^. In this study, we performed a series of experiments to fabricate a gold nanotip by electrochemical etching using a 37% HCl solution diluted with 99.9% anhydrous ethanol to examine the effect of the HCl concentration on the fabricated tip shape. From a statistical study of tips fabricated under various HCl concentrations, we found the optimal condition that produces a higher yield or sharper tip apex. Using electrochemically etched tips, we also obtained TERS images of an atomically thin tungsten disulfide (WS_2_) monolayer grown by chemical vapor deposition (CVD).

## Results and Discussion

### Gold nanotip fabrication by using electrochemical etching method

**Figure 1:**
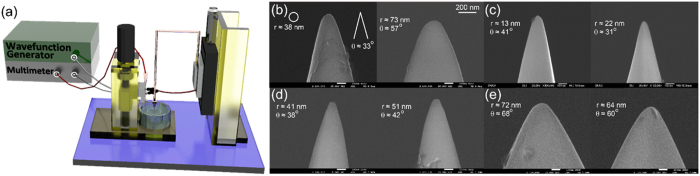
(**a**) Schematic diagram of the homemade electrochemical etching setup. A gold wire is connected to the positive pole (anode) of an arbitrary waveform generator to attract chloride ions and to initiate gold ionization. A platinum loop is connected to the negative pole (cathode) of the wave function generator. (**b**–**e**) SEM images of the etched gold tips fabricated with various concentrations of ethanol and HCl solution. (**b**) Only HCl solution. (**c**) HCl solution:ethanol = 3:1, (**d**) 2:1, and (**e**) 1:1 (Scale bar of right upper applies to all SEM images in **b**–**e**).

The etched gold tips fabricated using HCl and ethanol solutions with various concentration ratios were observed using a scanning electron microscope (SEM) to examine the *R*_c_ value and cone angle of the tip apex. Figure 1a shows a schematic diagram of the electrochemical etching method (see the Methods section). SEM images of representative tips fabricated using four concentration ratios are shown in Fig. 1b–e (HCl solution only and HCl:ethanol = 3:1, 2:1, and 1:1). The tips fabricated in HCl alone were rather blunt, having *R*_c_ values of 38 and 73 nm and marginal cone angles of 33° and 57°, respectively. At a lower HCl concentration (3:1 volume ratio), *R*_c_ decreases significantly to 13 and 22 nm, and then increases slowly as the HCl concentration decreases further, as can be seen for the 2:1 and 1:1 volume ratios (parts d and e, respectively, in Fig. 1), where *R*_c_ is large (72 and 64 nm) in the latter case. The cone angle of the tips does not seem to change significantly with decreasing HCl concentration except at the 1:1 volume ratio, which yielded large conical angles of 60° and 68°. These figures show that the effect of the HCl concentration on the tip shape cannot be easily determined because other uncontrollable conditions such as the temperature fluctuation of the solution, humidity of the laboratory, and microscopic fluctuation of HCl concentration in the etchant may affect the electrochemical etching process.

To statistically rule out the effects of such uncontrollable conditions, we fabricated ~100 tips at a fixed volume ratio. To obtain meaningful statistical parameters, we first excluded those tips that showed clear failure of the etching process, having an irregularly shaped apex or an *R*_c_ value larger than 100 nm (Figure S2). On the other hand, the tips fabricated successfully regardless of the etching conditions can be used to perform the TERS experiment (Figure S3). Table 1 summarizes the success rate, average *R*_c_ value, and cone angle for different HCl concentrations. The success rate is ~50% at the highest HCl concentration and increases gradually with decreasing HCl concentration, reaching 67% at a 2:1 volume ratio. However, the success rate drops suddenly at the lowest HCl concentration (1:1 volume ratio). This drop may be attributed to a reduced etching ratio, which potentially causes the gold wire immersed in the solution to suddenly fall away owing to gravitational forces, which may tear the gold wire in the etching region and ultimately introduce either an irregular apex shape or a blunt end. This scenario is also supported by the fact that the current drop was slower than that at other concentrations. Further, in Table 1, *R*_c_ decreases gradually as the HCl concentration decreases. At the highest concentration (HCl only), the average *R*_c_ value was 53.7 nm, and *R*_c_ shows a minimum of 21.7 nm at the lowest concentration. This trend can be attributed to a modification of the etching speed with variation in the HCl concentration, and corresponding variation in the shape of the meniscus formed at the upper surface of the solution close to the gold wire (Figure S5). At high concentrations, a deep dimple may be formed owing to the rapid etching speed (Figure S5). In this case, the area where active etching has occurred becomes narrower because the rise of the solution due to capillary force is restricted by the deep dimple formation, which introduces more accelerated etching rate at the neck of the dimple. When the diameter of the remaining gold wire becomes too small to support the mass of wire immersed in the solution, this region would be pulled by gravitational forces to introduce further narrowing, which acts positively to form a smaller apex. However, an accelerated etching speed may prevent this process, and simply *cutting* the wire will produce a relatively large *R*_c_. At low concentrations, however, a shallower dimple is formed because of the low etching rate, which causes the meniscus to rise higher, in contrast to that at a higher concentration. In this case, a relatively low etching rate can be maintained until the moment of apex formation. Hence, the pulling process may dominate, which consequently enables a smaller *R*_c_. This scenario agrees well with the discussion of the sudden drop in the success rate at the lowest concentration: at a much reduced etching rate, pulling of the gold wire frequently breaks the neck of the dimple rather than forming a sharp apex.

These observations imply that there should be an optimal HCl concentration: gold wire tends to be cut when the concentration is too high and to fall away when it is too low. Our observation suggests that the volume ratio of 37% HCl solution and 99.9% ethanol should be 3:1 for obtaining a sufficiently small *R*_c_ of 34 nm at a sufficiently high success rate of 59%.

### Tip-enhanced Raman scattering imaging

**Figure 2:**
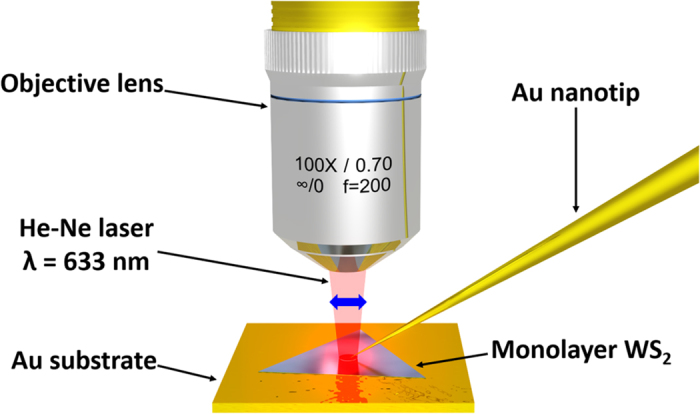
Schematic diagram of tip-enhanced Raman spectroscopy. Laser light having wavelength of 633 nm and output power of ~1 mW is focused to the monolayer WS_2_ on a gold substrate by using an objective lens with a magnification of 100× and a numerical aperture of 0.7. The blue arrow indicates a polarization direction of the laser.

**Figure 3:**
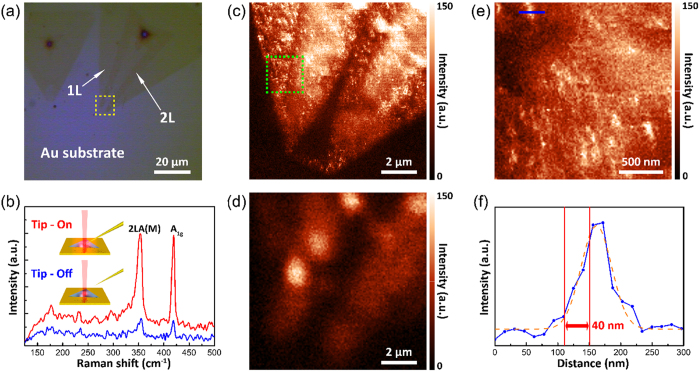
TERS spectra and images of mono/bilayer WS_2_ flake when applying a tip fabricated using a solution having 3:1 volume ratio of HCl and ethanol solutions. (**a**) Optical microscope image of the sample. (**b**) TERS spectrum (“Tip-on”, red curve) and confocal Raman spectrum (“Tip-off”, blue curve) measured at the same position on the sample. (**c**) TERS image of A_1g_ peak area intensity (Yellow dashed box in (**a**) indicates TERS mapping area.). (**d**) Confocal Raman scattering image of A_1g_ peak area intensity at the same position in (**c**). (**e**) Zoomed-in TERS image for a green dashed box in (**c**). (**f**) TERS intensity profile of the A_1g_ peak signal along the blue bar in (**e**) and the fitted Gaussian peak (orange dashed line). The TERS spatial resolution by applying edge response function was obtained as ~40 nm.

To test whether our tips can be successfully applied in TERS, we used fabricated a tip with small apex radius (Figure S6) and two-dimensional samples which indicate WS_2_ and graphene (Figure S7) in our TERS microscope, which was integrated with a scanning probe microscope, confocal microscope, and Raman scattering spectroscope (NTEGRA Spectra, NT-MDT). The excitation laser which is polarized along the tip-axis orientation (He-Ne laser, *λ* = 633 nm) is focused on the sample by an objective lens with a numerical aperture of 0.7 and a magnification of 100×, as shown in Fig. 2. The Raman scattering intensity and spectra are recorded using a charge-coupled detector (Andor) cooled to −70 °C and equipped with a spectrometer containing an 1800 grooves/mm grating blazed at 500 nm. The test specimen was monolayer WS_2_ grown on a SiO_2_/Si substrate by CVD. Monolayer WS_2_ shows strong photoluminescence (PL) emission from ~1.93 to ~1.96 eV because of a direct band gap^28^. When Raman scattering is measured using laser excitation at 633 nm, the PL background can be observed in the Raman spectrum of monolayer WS_2_^29^. To avoid the PL background using charge transfer to the metal substrate, monolayer WS_2_ flakes were transferred onto a gold substrate, which was fabricated by coating a SiO_2_/Si substrate with gold ~100 nm in thickness using thermal evaporation. Figure 3a shows an optical microscope image of monolayer WS_2_ flakes on the gold substrate, where bilayer WS_2_ grown arbitrarily during synthesis of monolayer WS_2_ is also visible. To determine the contrast in the Raman scattering signals and the Raman enhancement factor (*EF*) of TERS, the spectra were recorded at the same position (bilayer position) of the sample in TERS (tip-on) and normal Raman scattering (tip-off) measurement, as shown in Fig. 3b. The intensities of the A_1g_ peaks at ~419 cm^−1^ were compared to determine the contrast and *EF* of the TERS and confocal Raman scattering data. The contrast between the TERS and confocal Raman scattering intensities can be calculated using the amplitude ratio of the two peaks (*I*_Tip−on_ and *I*_Tip−off_). The *EF* value of TERS can also be calculated using the contrast, the radius of the laser spot (*R*_Laser_), and the *R*_c_ value of the tip (*R*_tip_). The equations for obtaining accurate contrast^30^ and *EF* values in TERS^31^ are defined as follows:


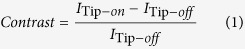



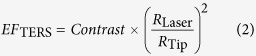


The value of the A_1g_ peak contrast is calculated to be ~5.2. In addition, the radii of the excitation laser spot and the tip apex are ~550 and ~18.2 nm, respectively. Applying these values to eq (2), we obtain an *EF*_TERS_ value of ~4,760. In addition, considering that the Raman scattering cross section is proportional to the fourth power of the electric field, we can also obtain an electric field enhancement factor of ~8.3, which is comparable to previously reported experimental results and theoretical predictions^6^,^14^. However, in practice, *EF*_TERS_ value have to be smaller than the aforementioned value because of the enlargement of the tip field due to inclination of the tip^32^. Including this effect, actual *EF*_TERS_ was obtained as ~3,370 when assuming tip inclination angle is 45°. Further, the analyses for a spatial resolution, which is related with TERS and confocal Raman scattering were carried out by showing a huge difference in the spatial resolution, as shown in Fig. 3c,d. Owing to the relatively weak Raman scattering signals and the diffraction-limited spatial resolution (Fig. 3d), it is difficult to distinguish the monolayer region and grain boundaries in the sample. In contrast to the confocal Raman scattering image in Fig. 3d, the TERS image in Fig. 3c shows the monolayer sample, boundaries between the monolayer and bilayer, and grain boundaries in monolayer WS_2_. To measure the spatial resolution, we acquired another TERS image with a step size of 15 nm, as shown in Fig. 3e. The TERS intensity profile of the A_1g_ peak along the blue bar in Fig. 3e represents the TERS spatial resolution, which reaches ~40 nm, as shown in Fig. 3f 1,33. The high spatial resolution of TERS beyond the diffraction limit is ~14 times better than that of confocal Raman spectroscopy.

**Figure 4:**
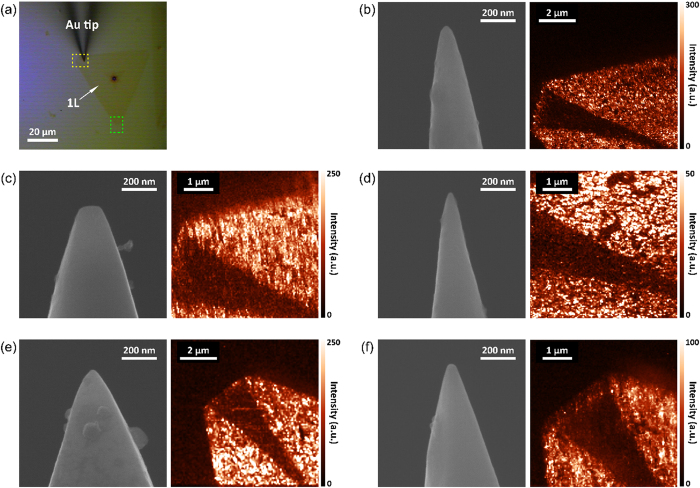
TERS images of A_1g_ peak area intensity of monolayer WS_2_ obtained by using several tips fabricated under the 3:1 volume ratio condition. (**a**) Optical microscope image of the tip approaching a sample. (**b**–**d**) Used tips and TERS images measured at a yellow dashed box in (**a**). (**e**, **f**) Used tips and TERS images measured at a green dashed box in (**a**).

To confirm whether other tips fabricated under the optimal condition can be used to obtain TERS images, several tips etched under the 3:1 volume ratio condition were also used to conduct TERS imaging. Figure 4a shows an optical microscope image of a monolayer WS_2_ flake on the gold substrate as the tip approaches the sample. Using different tips fabricated under the same condition, we successfully obtained various TERS images, as shown in Fig. 4b–f, even though radii and cone angles are slightly different each other. As shown in these figures, image qualities are different for various tip shape. For example, the image taken by rather blunt tip (~76 nm) depicted in Fig. 3c shows much reduced resolution as comparing with the others. These results indicate that the tip fabricated under a 3:1 volume ratio of HCl solution and ethanol, which is suggested as an optimal condition, can be used to achieve greatly enhanced Raman scattering signals and high spatial resolution in Raman scattering images.

## Conclusions

We determined the optimal composition of the HCl solution for fabrication of gold nanotips by electrochemical etching. We found that the optimum volume ratio of HCl and ethanol solution for obtaining a sufficiently small tip apex at an acceptable success rate was 3:1. We also demonstrated successful TERS spectroscopy for monolayer and bilayer WS_2_ flakes by using tips fabricated under this condition. We achieved a contrast of 5.2 and *EF*_TERS_ values as high as 4,760 when using an 18.2 nm radius of curvature. Further, we also obtained a TERS image with a high spatial resolution of 40 nm, which is beyond the diffraction limit. We suggest that our method can contribute to high efficiency production of nanotips that are suitable for high efficiency TERS and also high resolution TERS imaging.

## Methods

### Electrochemical etching process

The gold nanotips were prepared using a homemade electrochemical etching system as shown in Fig. 1a. In this system, the gold wire (Nilaco Co.) to be etched is connected to a positive pole, and a platinum ring (Nilaco Co.) is connected to the negative pole of an arbitrary waveform generator, which supplies a series of pulsed electric biases with a peak amplitude of 3.5 V, a background value of −25 mV, a frequency of 300 Hz, and a 20% duty cycle. This pulsed bias is expected to result in a relaxation time that allows the system to eliminate bubbles^21^ and any chemical instability caused by a violent reaction. The anode attracts anions such as chloride, which are generated near the cathode, to ionize the gold. As gold ionization progresses, the color of the HCl solution mixed with ethanol gradually changes from colorless and transparent to yellow because of precipitation of gold chloride (Figure S1). In our etching process, the platinum ring was first cleaned by immersing it in 36% HCl solution (Duksan) for 5 min, followed by rinsing with acetone, ethanol, deionized water, and isopropyl alcohol. To further remove any remaining chemicals, the ring was sonicated for 5 min. This ring was immersed in 37% HCl solution (Sigma-Aldrich) at an immersion depth of ~2 mm. A gold wire having a diameter of 250 μm was submerged in the same solution at a depth of ~0.5 mm. To investigate the effect of the HCl concentration on the etching process, four solutions were prepared by mixing pure 37% HCl solution and 99.9% anhydrous ethanol (Duksan) in different volume ratios (HCl only, 3:1, 2:1, and 1:1 for HCl: ethanol). An electrical bias with the properties described above was applied to the gold wire until the etching process was complete. A multimeter was used to monitor the current to assess the completion of the etching process. Such etching processes are usually self-terminating, and termination is manifested as a rapid decrease in current.

## Additional Information

**How to cite this article**: Lee, C. *et al*. Tip-Enhanced Raman Scattering Imaging of Two-Dimensional Tungsten Disulfide with Optimized Tip Fabrication Process. *Sci. Rep.* **7**, 40810; doi: 10.1038/srep40810 (2017).

**Publisher's note:** Springer Nature remains neutral with regard to jurisdictional claims in published maps and institutional affiliations.

## Supplementary Material

Supplementary Information

## Figures and Tables

**Table 1:** Statistics for apex radius and cone angle of gold nanotips corresponding to the composition of HCl and ethanol solution (Criterion of success is tip having apex radius below 100 nm).

HCl: Ethanol Ratio	Success Rate (%)	Radius of Curvature (nm)		Cone Angle (°)	
		Average	Deviation	Average	Deviation
HCl only	49.1	53.7	29.1	54.9	16.7
3: 1	58.8	34.4	21.4	35.2	10.4
2: 1	66.7	43.0	21.6	68.5	29.2
1: 1	21.7	21.2	12.1	55.9	26.4

## References

[b1] Chi Chen N. H. S. K. A 1.7 nm resolution chemical analysis of carbon nanotubes by tip-enhanced Raman imaging in the ambient. Nat. Commun. 5, doi: 10.1038/ncomms4312 (2014). 24518208

[b2] Kumar N., Stephanidis B., Zenobi R., Wain A. J. & Roy D. Nanoscale mapping of catalytic activity using tip-enhanced Raman spectroscopy. Nanoscale 7, 7133–7137, doi: 10.1039/c4nr07441f (2015). 25699648

[b3] Schmidt S. . Adiabatic nanofocusing on ultrasmooth single-crystalline gold tapers creates a 10-nm-sized light source with few-cycle time resolution. ACS Nano 6, 6040–6048, doi: 10.1021/nn301121h (2012). 22681506

[b4] Xue J. . Scanning tunnelling microscopy and spectroscopy of ultra-flat graphene on hexagonal boron nitride. Nat Mater 10, 282–285, doi: http://www.nature.com/nmat/journal/v10/n4/abs/nmat2968.html#supplementary-information (2011). 21317900 10.1038/nmat2968

[b5] Tapaszto L. . Breakdown of continuum mechanics for nanometre-wavelength rippling of graphene. Nat Phys 8, 739–742, doi: http://www.nature.com/nphys/journal/v8/n10/abs/nphys2389.html#supplementary-information (2012).

[b6] Park D. J. . Strong field acceleration and steering of ultrafast electron pulses from a sharp metallic nanotip. Phys Rev Lett 109, doi: 10.1103/PhysRevLett.109.244803 (2012). 23368330

[b7] Ropers C., Solli D. R., Schulz C. P., Lienau C. & Elsaesser T. Localized multiphoton emission of femtosecond electron pulses from metal nanotips. Phys Rev Lett 98, doi: 10.1103/PhysRevLett.98.043907 (2007). 17358773

[b8] Schenk M., Krüger M. & Hommelhoff P. Strong-field above-threshold photoemission from sharp metal tips. Phys Rev Lett 105, doi: 10.1103/PhysRevLett.105.257601 (2010). 21231628

[b9] Zhang R. . Chemical mapping of a single molecule by plasmon-enhanced Raman scattering. Nature 498, 82–86, doi: 10.1038/nature12151 http://www.nature.com/nature/journal/v498/n7452/abs/nature12151.html#supplementary-information (2013). 23739426

[b10] Rang M. . Optical Near-Field Mapping of Plasmonic Nanoprisms. Nano Lett. 8, 3357–3363, doi: 10.1021/nl801808b (2008). 18788789

[b11] Johnson T. W. . Highly Reproducible Near-Field Optical Imaging with Sub-20-nm Resolution Based on Template-Stripped Gold Pyramids. ACS Nano 6, 9168–9174, doi: 10.1021/nn303496g (2012). 22938087

[b12] Böhmler M., Wang Z., Myalitsin A., Mews A. & Hartschuh A. Optical Imaging of CdSe Nanowires with Nanoscale Resolution. Angewandte Chemie International Edition 50, 11536–11538, doi: 10.1002/anie.201105217 (2011). 21997847

[b13] Park K. D. . Laser fabrication of gold nanoparticle clustered tips for use in apertureless near-field scanning optical microscopy. J. Nanosci. Nanotechnol. 14, 5961–5964, doi: 10.1166/jnn.2014.8294 (2014). 25936036

[b14] Cançado L. G., Hartschuh A. & Novotny L. Tip-enhanced Raman spectroscopy of carbon nanotubes. J. Raman Spectrosc. 40, 1420–1426, doi: 10.1002/jrs.2448 (2009).

[b15] Wang P. . Reversible Defect in Graphene Investigated by Tip-Enhanced Raman Spectroscopy. Proc SPIE Int Soc Opt Eng 7, 555–561, doi: 10.1007/s11468-012-9342-8 (2012).

[b16] Vantasin S. . Tip-Enhanced Raman Scattering of the Local Nanostructure of Epitaxial Graphene Grown on 4H-SiC (0001 ¯). The Journal of Physical Chemistry C 118, 25809-25815, doi: 10.1021/jp508730y (2014).

[b17] Farshid Pashaee F. S. Giovanni Fanchini and François Lagugné-Labarthet. Tip-enhanced Raman spectroscopy of graphene-like and graphitic platelets on ultraflat gold nanoplates. Phys. Chem. Chem. Phys. 17, 21315–21322, doi: 10.1039/C4CP05252H (2015). 25684162

[b18] Beams R., Cançado L. G., Oh S.-H., Jorio A. & Novotny L. Spatial Coherence in Near-Field Raman Scattering. Phys Rev Lett 113, 186101 (2014). 25396381 10.1103/PhysRevLett.113.186101

[b19] Kim P. . Efficient electrochemical etching method to fabricate sharp metallic tips for scanning probe microscopes. Rev. Sci. Instrum. 77, 103706, doi: http://dx.doi.org/10.1063/1.2358703 (2006).

[b20] Kharintsev S. S., Hoffmann G. G., Fishman A. I. & Salakhov M. K. Plasmonic optical antenna design for performing tip-enhanced Raman spectroscopy and microscopy. J Phys D 46, 145501 (2013).

[b21] Ren B., Picardi G. & Pettinger B. Preparation of gold tips suitable for tip-enhanced Raman spectroscopy and light emission by electrochemical etching. Rev. Sci. Instrum. 75, 837–841, doi: http://dx.doi.org/10.1063/1.1688442 (2004).

[b22] Libioulle L., Houbion Y. & Gilles J. M. Very sharp gold and platinum tips to modify gold surfaces in scanning tunneling microscopy. Journal of Vacuum Science &amp; Technology B 13, 1325–1331, doi: http://dx.doi.org/10.1116/1.587847 (1995).

[b23] Billot L., Berguiga L., de la Chapelle M. L., Gilbert Y. & Bachelot R. Production of gold tips for tip-enhanced near-field optical microscopy and spectroscopy: analysis of the etching parameters. The European Physical Journal–Applied Physics 31, 139–145, doi: 10.1051/epjap:2005049 (2005).

[b24] Kharintsev S. S., Rogov A. M. & Kazarian S. G. Nanopatterning and tuning of optical taper antenna apex for tip-enhanced Raman scattering performance. Rev. Sci. Instrum. 84, 093106, doi: http://dx.doi.org/10.1063/1.4822274 (2013). 24089815 10.1063/1.4822274

[b25] Williams C. & Roy D. Fabrication of gold tips suitable for tip-enhanced Raman spectroscopy. J Vac Sci Technol B Microelectron Nanometer Struct 26, 1761–1764, doi: 10.1116/1.2981078 (2008).

[b26] Park J., Hong T. S., Lee N. S., Kim K. B. & Seo Y. Viscosity dependence of electrochemical etching for gold tip fabrication. Curr. Appl. Phys. 11, 1332–1336, doi: http://dx.doi.org/10.1016/j.cap.2011.03.075 (2011).

[b27] Li M. . Electrochemical fabrication of silver tips for tip-enhanced Raman spectroscopy assisted by a machine vision system. J. Raman Spectrosc. 47, 808–812, doi: 10.1002/jrs.4898 (2016).

[b28] Kim M. S. . Biexciton Emission from Edges and Grain Boundaries of Triangular WS2 Monolayers. ACS Nano 10, 2399–2405, doi: 10.1021/acsnano.5b07214 (2016). 26758415

[b29] Zhao W. . Lattice dynamics in mono- and few-layer sheets of WS2 and WSe2. Nanoscale 5, 9677–9683, doi: 10.1039/C3NR03052K (2013). 23999910

[b30] Stadler J., Schmid T. & Zenobi R. Developments in and practical guidelines for tip-enhanced Raman spectroscopy. Nanoscale 4, 1856–1870, doi: 10.1039/C1NR11143D (2012). 22105888

[b31] Kumar N., Rae A. & Roy D. Accurate measurement of enhancement factor in tip-enhanced Raman spectroscopy through elimination of far-field artefacts. Applied Physics Letters 104, doi: 10.1063/1.4869184 (2014).

[b32] Pettinger B., Schambach P., Villagómez C. J. & Scott N. Tip-Enhanced Raman Spectroscopy: Near-Fields Acting on a Few Molecules. Annual Review of Physical Chemistry 63, 379–399, doi: 10.1146/annurev-physchem-032511-143807 (2012). 22263910

[b33] Liao M. . Tip-Enhanced Raman Spectroscopic Imaging of Individual Carbon Nanotubes with Subnanometer Resolution. Nano Lett. 16, 4040–4046, doi: 10.1021/acs.nanolett.6b00533 (2016). 27348072

