# Supplementary material for: Tip-Enhanced Raman Scattering Imaging of Two-Dimensional Tungsten Disulfide with Optimized Tip Fabrication Process
Source: Sci Rep. 2017 Jan 13;7:40810. doi: 10.1038/srep40810 (PMC5234014; doi:10.1038/srep40810)
Supplement: Supplementary Information [file srep40810-s1.pdf]

Supplementary Information

Tip-Enhanced Raman Scattering Imaging of Two-Dimensional Tungsten Disulfide with Optimized Tip Fabrication Process

Chanwoo Lee[1], Sung Tae Kim[1,2], Byeong Geun Jeong[1], Seok Joon Yun[1,2], Young Jae Song[3,4], Young Hee Lee[1,2,4], Doo Jae Park[5,*], and Mun Seok Jeong[1,2,*]

[1]Department of Energy Science, Sungkyunkwan University (SKKU), Suwon 16419, Republic of Korea

[2]Center for Integrated Nanostructure Physics, Institute for Basic Science (IBS), Suwon 16419, Republic of Korea

[3]SKKU Advanced Institute of Nanotechnology (SAINT), Sungkyunkwan University, Suwon 16419, Republic of Korea

[4]Department of Physics, Sungkyunkwan University, Suwon 16419, Republic of Korea

[5]Department of Physics, Hallym University, Hallymdaehakgil 1, Chuncheon 24252, Republic of Korea

*doojaepark@hallym.ac.kr; mjeong@skku.edu

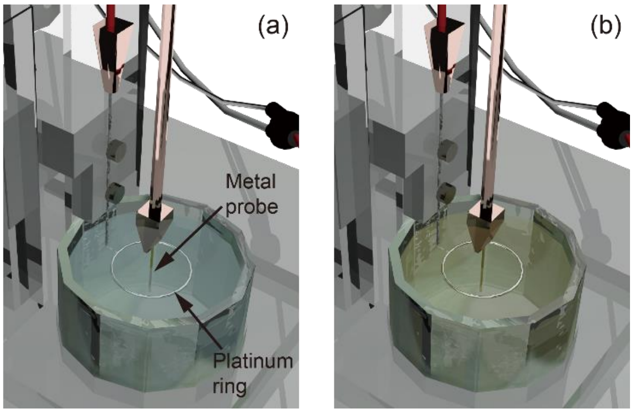

Figure S1. Illustrations of apparatus used in the electrochemical etching process (a) at the beginning of etching and (b) in the middle of the etching process. A gold wire immersed in HCl solutions having various concentrations is electrochemically etched by applying a series of pulsed biases having a maximum voltage of 3.5 V with a base voltage of −25 mV and a 20% duty cycle with a frequency of 300 Hz. During electrochemical etching, the solution becomes yellowish owing to dissolution of gold, as illustrated in (b).

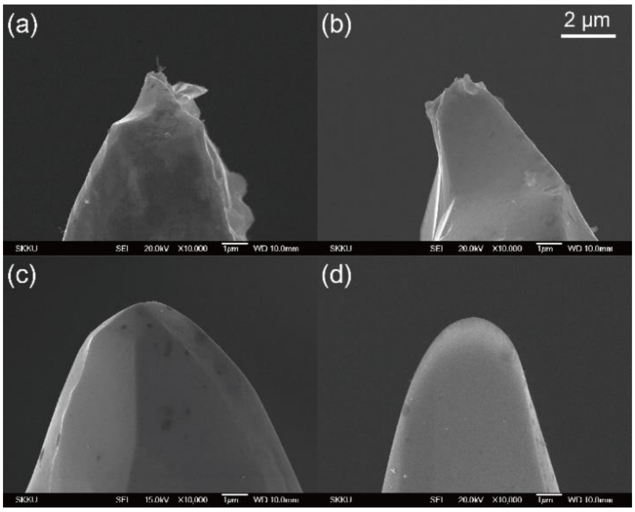

Figure S2. Gold tips indicating obvious failure of etching process in etching of ~100 tips for statistical study. These tips have (a, b) irregular and rough shapes or (c, d) a radius of curvature exceeding 100 nm.

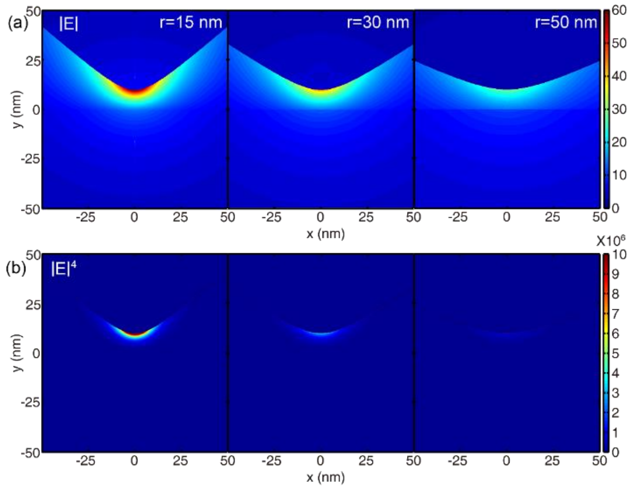

Figure S3. (a) Simulated electric field amplitudes near tip apexes with radii of curvature of 15, 30, and 50 nm, where tip-to-sample distance was maintained at 10 nm. (b) Fourth power of the electric field for simulating Raman scattering amplitudes in these tips.

A simulation based on analytic calculations was performed using a quasi-static approximation to visualize the electric field profile at the tip when it approached the sample. This model calculates the scalar potential by solving the boundary conditions at the tip surface as well as at the substrate.[1] To compare field enhancement and field confinement, R_c values of 15, 30, and 50 nm were applied to the tip, and the tip-to-sample distance was constant at 10 nm. The dielectric constant of gold was chosen as −10.7, assuming an incident laser wavelength of 633 nm,[2] and that of the substrate was set to 2.89, assuming a sapphire substrate. As shown in Figure S3a, the electric field is greatly enhanced at the tip apex for an R_c value of 15 nm and decreases slowly. The peak electric field strength for an R_c value of 15 nm was about twice that for an R_c value of 50 nm. In addition, to visualize the Raman enhancement, the fourth power of the electric field is plotted in Figure S3b. Obviously, the Raman enhancement is much larger and its distribution becomes much narrower for an R_c value of 15 nm. This implies that the resolution of Raman images could be improved by using a sharper tip in TERS imaging.

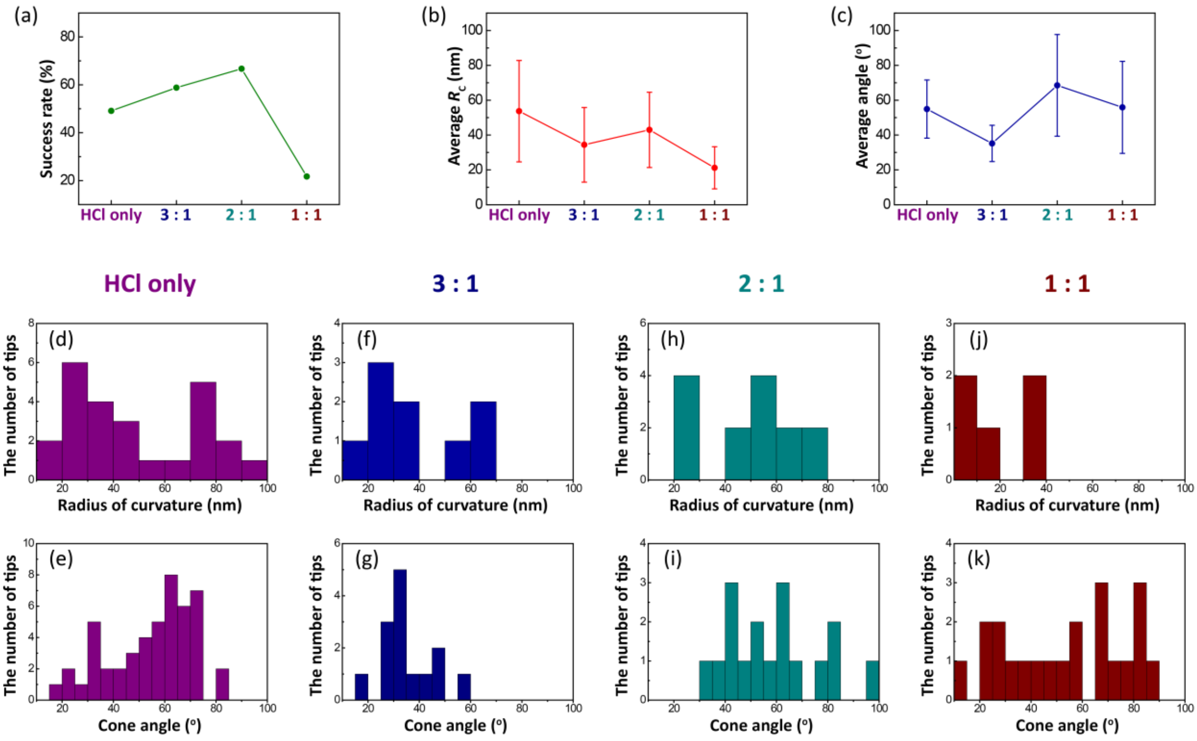

Figure S4. Statistics for the etched tips (a) Graph for the success rate under each condition. (b) Graph for the average radius of curvature under each condition and the error bars indicate the deviation. (c) Graph for the average cone angle under each condition and the error bars indicate the deviation. (d-k) Histograms for the number of tip versus radius of curvature and cone angle under four conditions.

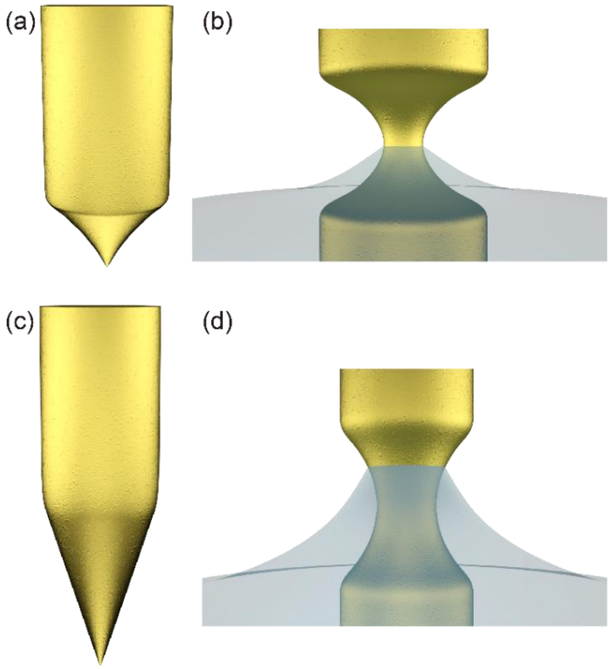

Figure S5. Schematic of etching speed and meniscus formation depending on concentration of HCl solution for (a, b) higher HCl concentration and (c, d) lower HCl concentration.

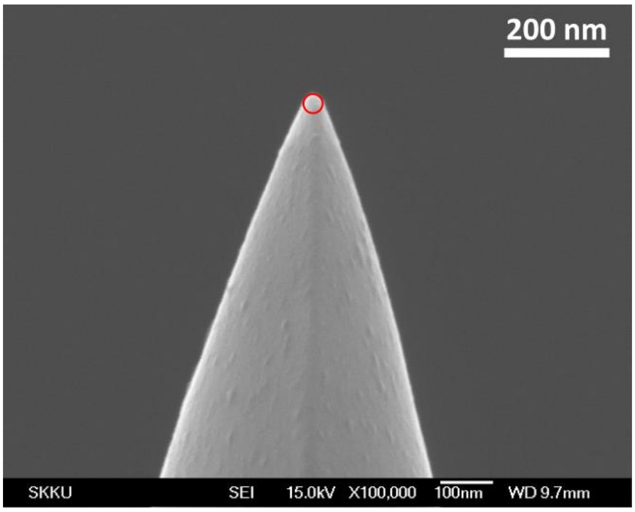

Figure S6. A tip fabricated using a 3:1 volume ratio of HCl solution and ethanol that were used to perform TERS imaging in Fig. 3.

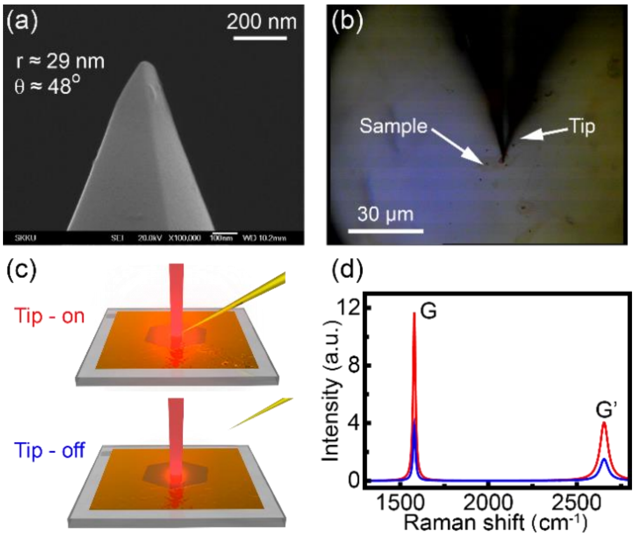

Figure S7. TERS signal of multilayer graphene obtained using a tip fabricated using a 3:1 volume ratio of HCl solution and ethanol. (a) SEM image of used tip. (b) Optical microscope image of the tip approaching a sample. (c) Schematic of tip retracted from the sample ("Tip-off") and approaching the sample ("Tip-on"). (d) Tip-enhanced Raman spectrum (Tip-on, red curve) and normal Raman spectrum (Tip-off, blue curve) measured at the same position on the sample.

References

1 Behr, N. & Raschke, M. B. Optical antenna properties of scanning probe tips: Plasmonic light scattering, tip-sample coupling, and near-field enhancement. *J. Phys. Chem. C* **112**, 3766-3773, doi:10.1021/jp7098009 (2008).

2 Johnson, P. B. & Christy, R. W. Optical Constants of the Noble Metals. *Physical Review B* **6**, 4370-4379 (1972).
